# P04.32. Acupuncture and heart rate variability: a systems level approach to understanding mechanism

**DOI:** 10.1186/1472-6882-12-S1-P302

**Published:** 2012-06-12

**Authors:** B Anderson, A Nielsen, B Kligler, D McKee

**Affiliations:** 1 Pacific College of Oriental Medicine, New York City, USA; 2 Beth Israel Medical Center, New York City, USA; 3 Albert Einstein College of Medicine, Yeshiva University, New York City, USA

## Purpose

Recent research has elucidated several different mechanisms for acupuncture. However the inter-relationship between these mechanisms and how acupuncture affects complex physiological systems is still not understood. Heart rate variability (HRV), the beat-to-beat fluctuations in the rhythm of the heart, results from the regulation of the heart by the autonomic nervous system (ANS). Low HRV is associated with increased risk of all-cause mortality and is a marker for a wide range of diseases. Coherent HRV patterns are associated with increased synchronization between the two branches of the ANS, and when sustained for long periods of time result in increased synchronization and entrainment between multiple body systems. This presentation is a systematic review of the clinical trials that have been undertaken examining the effect of acupuncture on HRV and the implications for HRV representing a systems level mechanism for acupuncture.

## Methods

The literature was reviewed using Medline, Science Citation Index, Cochrane (Database of Systematic Reviews and Central Register of Controlled Trials), the New England School of Acupuncture library databases, cross-reference of published data, personal libraries and Chinese medicine textbooks.

## Results

Results from randomized placebo controlled trials strongly suggest that acupuncture can improve HRV, especially when acupuncture is delivered in clinically valid dosages to subjects with a medically diagnosed condition and with the inclusion of an inert placebo control.

## Conclusion

There is sufficient evidence in the literature to support the conclusion that acupuncture improves HRV. Acupuncture may function by mediating global physiological regulation through improvement of HRV and synchronization of the two branches of the ANS. As a complex intervention, such a view of acupuncture mechanism is conceptually aligned with systems and complexity theory and is more compatible with traditional East Asian medical theory.

